# Correction: Unraveling the structural and chemical features of biological short hydrogen bonds

**DOI:** 10.1039/c9sc90176k

**Published:** 2019-08-20

**Authors:** Shengmin Zhou, Lu Wang

**Affiliations:** a Department of Chemistry and Chemical Biology , Institute for Quantitative Biomedicine , Rutgers University , Piscataway , NJ 08854 , USA . Email: lwang@chem.rutgers.edu

## Abstract

Correction for ‘Unraveling the structural and chemical features of biological short hydrogen bonds’ by Shengmin Zhou *et al., Chem. Sci.*, 2019, DOI: ; 10.1039/c9sc01496a.



## 

The authors regret that in the original article funding information was inadvertently omitted in the Acknowledgements. The authors gratefully acknowledge the National Science Foundation through the award CHE-1904800.

The Royal Society of Chemistry apologises for these errors and any consequent inconvenience to authors and readers.

